# ‘Having a Quiet Word’: Yarning with Aboriginal Women in the Pilbara Region of Western Australia about Mental Health and Mental Health Screening during the Perinatal Period

**DOI:** 10.3390/ijerph16214253

**Published:** 2019-11-01

**Authors:** Emma Carlin, David Atkinson, Julia V Marley

**Affiliations:** The Rural Clinical School of Western Australia, The University of Western Australia, Broome, WA 6725, Australia

**Keywords:** Aboriginal, indigenous, perinatal depression and anxiety, screening, clinical care, qualitative research, perinatal mental health

## Abstract

Despite high rates of perinatal depression and anxiety, little is known about how Aboriginal women in Australia experience these disorders and the acceptability of current clinical screening tools. In a 2014 study, the Kimberley Mum’s Mood Scale (KMMS) was validated as an acceptable perinatal depression and anxiety screening tool for Aboriginal women in the Kimberley region of Western Australia. In the current study, we explored if it was appropriate to trial and validate the KMMS with Aboriginal women in the Pilbara. Yarning as a methodology was used to guide interviews with 15 Aboriginal women in the Pilbara who had received maternal and child health care within the last three years. Data were analysed thematically, the results revealing that this cohort of participants shared similar experiences of stress and hardship during the perinatal period. Participants valued the KMMS for its narrative-based approach to screening that explored the individual’s risk and protective factors. While support for the KMMS was apparent, particular qualities of the administering health care professional were viewed as critical to the tool being well received and culturally safe. Building on these findings, we will work with our partner health services in the Pilbara to validate the KMMS with Pilbara Aboriginal women.

## 1. Introduction

Positive mental health and wellbeing is increasingly recognised as central to a person’s ability to achieve and maintain other health outcomes [1,2,3]. In the perinatal period, this includes but is not limited to optimal use of health care services [4]. The enduring disparity in Aboriginal maternal and child health outcomes [5,6,7], the high rates of Aboriginal perinatal depression [4,8,9,10,11], and the known consequences of untreated perinatal mental health disorders (including poorer psychological and development outcomes for children and reduced quality of life for women) [12,13], continues to position Aboriginal women’s perinatal mental health as a matter of national significance [14].

In Australia, national clinical guidelines [15] recommend routine use of the the Edinburgh Postnatal Depression Scale (EPDS) [16] to identify and respond to perinatal mental health concerns. The EPDS, validated in 1987 [16], represented a significant step in advancing a routine and ‘effective’ clinical screening tool for perinatal depression and anxiety. However, growing recognition of the importance of a woman’s context in the diagnosis of perinatal mental health concerns has led to the development of broader psychosocial assessments, such as the Antenatal Risk Questionnaire (ANRQ) to be used in addition to the EPDS [17,18,19]. Psychosocial tools are designed to explore more about the woman’s individual life story to better assist the health care professional in determining a woman’s overall risk of perinatal mental health disorders and the optimal pathways for treatment and support. Given concerns around the suitability of the EPDS with Aboriginal women [8,20,21,22] and the current rates of under screening [4,23], psychosocial screening represents a new and perhaps better way for health care professionals to engage with Aboriginal women around their perinatal mental health [20].

Over a hundred Aboriginal women from the Kimberley region, in partnership with health care professionals, designed the Kimberley Mum’s Mood Scale (KMMS) as an alternative approach to screening Aboriginal women for perinatal depression and/or anxiety [24]. Part 1 of the KMMS is an adapted version of the EPDS using Kimberley language and graphics (Supplementary Materials 1). Part 2 of the KMMS is a template for a psychosocial ‘yarn’ [25,26,27], a participant-led narrative based exploration of a woman’s risk and protective factors. The yarn is centred on seven domains (Supplementary Materials 2) that were identified by Aboriginal women and the existing literature [28] as important components in understanding a woman’s mental health and wellbeing. The KMMS Kimberley validation study [8] demonstrated that the yarning process (KMMS Part 2) fostered a rapport between the woman and her health care professional that enabled the woman to share details of her life. KMMS Part 2 uses open ended questions (Supplementary Materials 3) to explore the woman’s unique context and to inductively build her profile of risk while reflecting on her protective factors and her resiliency [29]. This is in contrast to other perinatal screening tools, such as the EPDS and the ANQR, that use closed questions with numerical scores to determine risk likelihood. In addition, neither the ANQR nor the EPDS promote a strengths-based [30] exchange between the woman and her health care professional during the screening process. The KMMS approach to screening was highly valued and the Kimberley validation study [8] found that Aboriginal women and their health care professionals identified the KMMS as acceptable and culturally appropriate.

This study, ‘Having a quiet word’, reflects on the yarning-based consultation process we undertook with Aboriginal women in the Pilbara region of Western Australia to explore the acceptability of the KMMS as a feature of their perinatal health care. This includes an understanding of how Aboriginal women in the Pilbara experience perinatal mental health issues, what Aboriginal women in the Pilbara value in terms of engagement and support for their perinatal mental health and women’s overall perceptions of trialling the KMMS within Pilbara health services. 

## 2. Materials and Methods 

### 2.1. Setting the Scene

The Pilbara is a vast, remote and sparsely populated geographical area in northern Western Australia. The Pilbara is home to a globally significant resource industry, which elevates the region’s population to around 60,000 people. Amongst this demography is an Aboriginal population of around 16% who, at a population level, experience significant and longstanding health disparities compared to the non-Aboriginal population in the region [31].

After the KMMS was validated in the Kimberley region, midwives from Mawarnkarra Health Service Aboriginal Corporation (Mawarnkarra), along with Western Australian Country Health Service (WACHS) staff, enquired about using the KMMS with Aboriginal women in the Pilbara. As Aboriginal people are culturally and linguistically diverse, we could not assume that the KMMS (developed and validated in the Kimberley) would be valid and/or acceptable for Aboriginal women in a separate geographic area, namely the Pilbara. Initial conversations with Pilbara midwives and health services developed into a partnership project between the Rural Clinical School of Western Australia (RCSWA), Mawarnkarra and WACHS-Pilbara to validate the KMMS amongst a sample of Pilbara women. This paper discusses phase one of the project, the initial consultations with Aboriginal women in the Pilbara about the KMMS as a possible feature of routine care. Further phases of the study, including the validation, will be reported on in separate papers. 

Prior to the research commencing in the Pilbara, RCSWA spent two years liaising and developing relationships with local partners and health services. Despite attempts and available funding, we were unable to secure a Pilbara Aboriginal co-researcher to work alongside us during the design, interviewing and analysis of this study. This was, in part, due to the existing workloads and other responsibilities of Aboriginal people in the Pilbara who were interested in and supportive of the project. In the absence of an Aboriginal co-researcher, our relationship with Aboriginal staff at recruitment sites and the Pilbara Aboriginal Health Planning Forum (PAHPF) was crucial in obtaining feedback on the research design, approach and analysis, and ensuring that the study was undertaken in a culturally safe way. This is discussed further below. 

### 2.2. Research Approach: Yarning as Methodology

This study employed the qualitative methodology of yarning [25] to elicit a participant-led narrative exploring experiences and perceptions of perinatal mental health. Yarning is recognised as a culturally sensitive approach to research with Aboriginal peoples [25,26,27,32]. The methodology is framed on the development of rapport between Aboriginal people and the researcher that allows for a respectful and robust exploration of a select research topic through the well-known Aboriginal communication process of yarning [25,27,32]. 

Table 1 Yarning as methodology.

### 2.3. Recruitment

Women were purposively recruited from play groups (n = 8), mums and bubs groups (n = 2), community arts centres (n = 2) or via introductions from maternal and/or child health care professionals (n = 3). Inclusion criteria for recruitment included Aboriginality and a recent experience of receiving perinatal care in the Pilbara region. ‘Recent’, for the purpose of this study, was defined as within a period of three years. 

During recruitment, the researcher talked to and provided women with a copy of the Participant Information Form, outlining the purpose and approach of the study. The researcher talked to the participants about the sensitive nature of the research yarn and that if at any stage the woman felt distressed or upset, the yarn would be stopped without any negative consequences. The woman and the researcher talked about relevant support options in the event of a woman becoming upset, noting that formal social and emotional wellbeing support may not be available or desired.

In total fifteen (15) perinatal Aboriginal women from three towns and two remote communities in the Pilbara were recruited and subsequently interviewed. The women were at various stages in the perinatal period. The group consisted of pregnant women (n = 4), women who had a child under the age of three (n = 6), and women who were both pregnant and had a child under the age of three (n = 5). The women were aged between 18 and 42 years and all identified as having grown up in various towns or communities within the Pilbara region. 

### 2.4. Interview Process 

The research topic yarn [25] included three pillars of enquiry: (1) perceptions and experiences of perinatal depression and anxiety, and perceptions of prevalence of perinatal depression and anxiety in the Pilbara; (2) experiences of perinatal depression and anxiety screening at a primary health care service; and (3) acceptability of the KMMS for Aboriginal women in the Pilbara. To explore these areas, the researcher used active listening techniques, respected long silences and infrequently prompted using open-ended questions. All participants were provided with a copy of the KMMS Part 1 and Part 2 and a copy of the EPDS during the interview.

Nine out of the 15 women consented to audio recording their yarn. Seven women listened to their yarn directly after it took place. All chose to have the interviewer in the room while they listened. Prior to listening to their yarn, participants were told that they could stop the recording at any time if they wanted to retract or elaborate on any information collected during the yarn. 

Two women who consented to audio recordings but did not wish to listen to their recording were provided a copy of their transcripts within two days of the interview. One woman chose to have the interviewer read the transcript to her. She made no retractions but did elaborate on some areas. 

The participants who did not consent for an audio recording of their yarn were shown the notes collected at the end of the interview, and were offered the opportunity to add or retract information. All participants provided some additional direct quotes during the note review process.

Interviews ranged from 30 minutes to one hour, but the associated time of introducing the research, consenting, tending to children and reflecting on the data generated during the yarn meant that the interviewer spent on average 1.5 hours with each woman. 

### 2.5. Interview Analysis

The recorded interviews and field notes were transcribed by the researcher and then descriptively and iteratively coded using NVivo11 (QSR International). The coding categories were reviewed by the researcher and once coding was complete, preliminary themes were identified from the data and discussed with participants and staff from the recruitment sites. Based on the review process, further thematic analysis was completed by the researcher with three multifaceted themes emerging. An interim plain language results paper was developed which included all verbatim quotes being considered for inclusion in the final publication. On subsequent field trips to the Pilbara, the researcher presented the plain language summary to the data collection sites. Participants and key Aboriginal staff were provided with an opportunity to provide feedback on the thematic areas and the use of direct quotes. Additionally, a later draft of the manuscript was sent to the PAHPF for review and endorsement. 

### 2.6. Ethics

This study is aligned to the National Health and Medical Research Council (NHMRC) guidelines on ethical conduct in Aboriginal and Torres Strait Islander health research [33]. The study was endorsed by the PAHPF prior to receiving formal ethics approval from the Western Australian Aboriginal Health Ethics Committee (Project 781). Informed written consent was obtained from participants prior to participation in the study and the methodology employed allowed participants to re-confirm/withdraw consent at several stages prior to publication.

## 3. Results

### 3.1. Theme One: A lived Experience 

Seven of the fifteen women spoke about their or a close family member’s experience with perinatal depression and/or anxiety. A further six women identified extreme and multiple stressors during their own perinatal period (violence, homelessness, drug and alcohol use, child protection intervention, traumatic birth outcomes, primary health problems) but did not talk about depression or anxiety. They talked about things being ‘hard’ or ‘stressful’.

*‘I was on my own for my pregnancy, I was excited but scared too you know. Her dad [*baby’s dad*] was in jail for drink driving. I stopped drinking for her but it was still hard, my dad and I don’t talk and my mother passed. I was sometimes stressing out’.*

(Participant 002)

The idea of not knowing how to talk or having the ‘right’ words was a common theme raised during the yarns. Participants spoke about being unfamiliar with the language of depression and anxiety.

*‘I never heard anyone say they are depressed, I hear them say they are ‘stressing out’, ‘going mad’. Sometimes we might use language words, but those are the ones mostly. We find it hard to talk about pain, I don’t know why, I think as Aboriginal people we like to laugh and talk for the good times but now everyone has a story of pain and we don’t know how to talk [*about it*]’.*

(Participant 010)

Participants spoke about the multiple stressors that many Pilbara Aboriginal women are negotiating while pregnant or having a small child. Violence, alcohol and drugs, homelessness, caring responsibilities, financial instability and not having family support were frequently mentioned as directly impacting on a woman’s wellbeing. Mothers leaving their babies with family members for extended periods of time or mothers who were not responsive to their baby’s needs (feeding, changing nappies, etc.) were identified by participants as suffering from ‘something serious’, although it was not always understood as perinatal depression and/or anxiety.

### 3.2. Theme Two: Connectedness and Support

Participants spoke about clinics and family both having a significant role in supporting women during the perinatal period. Five participants spoke about the support they received from intimate and/or family relationships and the ‘strength’ this gave them.

*‘Since my sister went through this [*perinatal depression*] we are all a lot more aware. We don’t, like, bang on about it, but I see they [*partner and mother*] ask me things in a way we never talked about before…. I feel supported, I think whatever happens I will be ok’.*

(Participant 008)

Several participants suggested that family do not always have the capacity to ‘help take the load off’, particularly families that are struggling with the effects of intergenerational trauma and/or intergenerational alcohol misuse. Six women talked about the importance of their relationship with their midwife. Home visiting, feeling cared about, ‘worried for’, and the continuity of care during pregnancy were the commonly identified elements of value in the relationship. Four of the six women who spoke about the importance of the relationship with their midwife had inaccessible or weak family support.

*‘My midwife is in* [name of Pilbara town] *but she is gone now. She was really good to me. I still call her. She made me feel it is ok to talk even when I didn’t know what I was going to say’.*

(Participant 001)

Religion was noted by a third of the participants (five women) as a strong protective factor for mental wellbeing. Religion was spoken about as a catalyst for a positive life change (e.g., finding religion and giving up drinking) or religion was discussed as an a priori protective factor (e.g., because I am a Christian my family don’t drink at my house). All five respondents who talked about religion stated that their religion gave them someone to talk too.

Support from formal counselling services was discussed by five participants. Two participants discussed their experience of counselling as a response to an acute stressor.

*‘My children saw all that violence with their father and then when my younger brother died [*by suicide*] the lights went out for me. I was pregnant and I took myself in to see the [*name of service*]. No one* [in the participant’s family] *was talking and I was starting to go mad. My other brothers, everyone is so broken from it and sometimes on the garri* [alcohol] *they might talk but it turns sour. So I tried it. The whitefella way [laughs]. It was alright, I was a bit thing* [shy] *but the lady was kind to me and, and, it was good to talk’.*

(Participant 011)

One participant spoke about counselling as a transformative experience.

*‘If you want to push through the shame* [of going to counselling] *you can… I learnt about myself. I got my kids back, I said fuck you to violent relationships. I wasn’t going to be like my mum and spent 20 years getting belted. I wanted to nurture my kids. I don’t think I had that nurture ‘. *

(Participant 003)

Two participants spoke about counselling ‘not being enough’, they both identified that for them, a cultural dimension to ‘healing’ was required. The counselling they had attended was unable to provide a cultural lens or dimension and ultimately that prevented both participants from seeing counselling as a longer-term therapeutic option. Therapeutic options that involved participants connecting with country were highly esteemed.

*‘I need something else, some other way to heal. Something with my old people and being on my ancestors land. The drugs and the violence get me down. I think the counsellor was good but I don’t know’…*

(Participant 014)

### 3.3. Theme Three: Yarning Safely

Two participants remembered being formally screened for perinatal depression and anxiety by their health care professional using the EPDS. A further five remembered their midwife asking about mood and wellbeing during the pregnancy. 

All participants, when asked, supported the notion of clinics asking all perinatal women about their mood. Participants stated that outside the clinic, these conversations may not happen even in families that are characterised by high levels of support. When shown the EPDS and the KMMS, screening via the KMMS was universally supported by the participants. The simplified language in Part 1 and the yarning component of Part 2 were identified as the reasons why the KMMS was the preferred screening tool. While support for the KMMS was apparent, several participants spoke about the ‘who, how and why’ of administering the KMMS as critical determinants in the tool being well received and viewed as culturally safe.

*‘Clinics need to talk these things through* [KMMS Part 2], *they really do, but they need to build up the friendship and trust… We are coping with so much loss. So much sadness…. You have to be really clear with the mum that this is for mum to help her stay strong and look after bub. It is not for DCP* [Department of Child Protection] *or anyone else, only the midwife and maybe a doctor’.*

(Participant 007)

Kindness and an ability to listen were highly valued when having a ‘quiet word’ with Aboriginal women about their mental health and wellness. Broad and gentle questions were seen as ‘safe’ or the ‘right’ approach to engaging women about their mental health and wellbeing. Participants warned that direct questions when asking about relationships, current stressors and childhood experiences could cause a woman to ‘clam up’ or disengage. Starting broadly and gently was seen as an opportunity for Aboriginal women to assess their health care professionals to understand if they were ‘judgemental’, ‘bossy’ or ‘worrying for something else’ (not interested in the woman’s story). Once a health care professional was deemed trustworthy, participants stated that most Aboriginal women would ‘share’ their story. 

All participants identified how strong and capable most Pilbara Aboriginal women are in managing their lives and their families despite the complexities they face. The majority of participants emphasised that a focus on their strength, resiliency or protective factors was important. Participants perceived this to have two benefits, first; helping women to reflect on how well they managed, and secondly, to assist the health care professional in understanding the woman as a whole person.

*‘Talking about the protective stuff, right, the stuff that keep us going, keeps us strong, that’s something. We are living this life the best way we can and for us to hear that. For clinic, my midwife to hear that. Now that is a powerful thing’.*

(Participant 009)

Participants emphasised the ‘building up’ of pressures or hardship over a woman’s life and identified how important it was to yarn. For several participants, this led to a direct questioning of the need to limit the Part 1 of the KMMS to a timeframe of the last week (Supplementary Materials 1). These participants stated that the timeframe was artificial and ultimately unhelpful in women engaging, often for the first time, in conversations about their mental health.

*‘You talk about things because they are important to talk about not cause they happened one week ago! It is a real whitefella way to start. It’s like you’re in or you’re out. You see that hey? Like what happens if it was a bit longer, then the lady might think oh no, it’s not important I won’t talk about that’.*

(Participant 015)

Aboriginal health care professionals were identified by around half of the participants as their preferred KMMS administrator. Talking to Aboriginal Health Workers or Aboriginal support staff was seen as less ‘shameful’, as Aboriginal staff would better understand a patient’s perspective and be less judgemental. Other participants identified a preference for an ‘outsider’, mostly their midwife or child health nurse. The preference for an outsider was related to a preconception of enhanced confidentiality and a desire to keep personal business ‘outside of the ears of community’. 

Participants talked about a critical need for education and resources tailored for Aboriginal women and their families. Participants stated that resources would be instrumental in helping women identify the signs and symptoms of perinatal mental health concerns, promote health-seeking behaviours and assure women that they are not alone. No participants reported being provided with meaningful information about mood and emotional wellbeing during the perinatal period. Clinics were identified as an important provider of education and repository of resources. Play groups were also identified as an important outreach site for local health services.

## 4. Discussion

The women in this study highlighted the critical and unique role that health care professionals have in engaging and supporting Aboriginal women’s mental health during the perinatal period. Health care professionals were often positioned as the sole broker of information about perinatal mental health. Additionally, most participants stated that outside of the clinical environment, conversations about their mental health and wellbeing were not occurring, even for women who had close and supportive intimate and familial relations. While these findings are aligned with other studies [4,6,9,11,34,35,36,37,38,39,40], this is the first example we could find in which Aboriginal women clearly and strongly identified their health care professionals (particularly midwives and Aboriginal Health Workers) as having a multifaceted role in supporting their perinatal mental health inclusive of engagement, education, screening and support. 

Women reported that their decision to engage with a health care professional was contingent on the personal characteristics of the health care professional (Aboriginality, non-Aboriginality, kindness) and the health care professional’s approach (yarning, non-judgemental, active listening). This is consistent with other qualitative Aboriginal mental health literature which found that Aboriginal people place high value on trusting, equitable relationships with health care professionals (including Aboriginal staff), in which the limits of confidentiality are clearly understood [41,42,43,44,45,46]. These studies evidence the importance of health care professionals engaging with patients to a level in which the health care professional can recognise and support the resiliency of the patient alongside the identification of the patient’s risk factors. Prominent in this literature is the emphasis on ‘yarning’ as a method of engaging Aboriginal people [41,42,44,45,46,47,48]. These key principles of patient engagement and care transcend the Aboriginal mental health space and are enshrined in Aboriginal-led understandings of culturally secure primary health care more broadly [49,50,51].

Throughout this study, many of the women’s narratives featured high levels of violence, abuse, grief and loss, which were deeply entwined with their concept of ‘wellbeing’. The women in this study valued clinical screening tools that allow for exploration of these and other significant areas of their life in a way that was gentle and provided them an opportunity to reflect on their strengths and protective factors. Current mainstream approaches to psychosocial screening, including the ANRQ [17], do not reflect these values. Instead the ANRQ adopts a deductive approach to screening in which predetermined areas of risk form a series of direct screening questions (Supplementary Materials 4). Responses to the questions are predefined quantitative responses and correspond to a risk rating. Deductive algorithm-based approaches, such as the ANRQ, are questionable for use with marginalised populations [52]. First there is the concern that marginalised populations are often not meaningfully accounted for in the development or validation of screening tools [44]. This appears to be the case for the ANRQ [13,45]. We argue that unless screening tools align with what Aboriginal women value [8,20,34,43], rates of under-screening [1,18] will persist, along with missed opportunities for early detection, treatment and support of Aboriginal women’s perinatal mental health. 

Effecting positive change in the perinatal mental health of Aboriginal mothers has untold positive net benefit on other maternal and child health outcomes. This study contributes to the evidence base that screening tools developed alongside Aboriginal women, such as the KMMS, are essential in screening Aboriginal women in a way that is meaningful to them. This study identifies that supporting Aboriginal women’s perinatal mental health requires an investment in maternal and child health staff (particularly Aboriginal Health Workers) to listen, yarn and celebrate the resiliency of a patient. Culturally safe screening practices also require a commitment by health services, Australian medical policy, and the associated Medicare Benefits Scheme to enable longer appointment times to ensure that a meaningful clinical investment can be made in Aboriginal women’s perinatal mental health.

As noted in the Methods section, data collection and analysis for this study was undertaken by a non-Aboriginal researcher. We acknowledge the limitations that may have arisen from data collection by a non-Aboriginal researcher; we also acknowledge that for some women, the ‘outsider’ status of the researcher was a factor in their willingness to participate. We sought input from a variety of Aboriginal stakeholders (including staff at recruitment sites and participants) in the analysis of the data to ensure a culturally secure interpretation of the data. 

## 5. Conclusions

These findings contribute to our understanding of how Aboriginal women perceive their own perinatal mental health. Particular to this study was an exploration of the scope and acceptability of health care professionals in engaging Aboriginal women around their perinatal mental health. Exploring what Aboriginal women in the Pilbara require from health services in terms of engagement and screening is an important precursor to advocating for their unique needs to be heard and upheld amongst the prevailing deductive approaches to perinatal mental health screening. We will continue to work with our partner health services in the Pilbara to validate and assess the user acceptability of the KMMS for Aboriginal women and their health care providers. 

## Figures and Tables

**Table 1:** Yarning as methodology. Steps used in ‘Having a quiet word’: yarning with Aboriginal women in the Pilbara Region of Western Australia about mental health and mental health screening in the perinatal period.

Step	Description
1	Introduction and brokerage of the researcher in the participant’s space
2	Deconstructing the research agenda
3	Establishing informed consent
4	The research topic yarn
5	Reviewing the yarn and re-confirming permission for future use of data
6	Reflecting, coding and analysis
7	Presentation of results to participants, key Aboriginal stakeholders and the Pilbara Aboriginal Health Planning Forum for review, feedback and revision
8	Publication of results and dissemination back to key stakeholders and participants

## Supplementary Materials

The following are available online at https://www.mdpi.com/1660-4601/16/21/4253/s1.
