# Supplementary material for: ‘Having a Quiet Word’: Yarning with Aboriginal Women in the Pilbara Region of Western Australia about Mental Health and Mental Health Screening during the Perinatal Period
Source: Int J Environ Res Public Health. 2019 Nov 1;16(21):4253. doi: 10.3390/ijerph16214253 (PMC6862568; doi:10.3390/ijerph16214253)
Supplement: Supplementary file 1 [file ijerph-16-04253-s001.zip › Supplementary Materials 1.pdf]

Kimberley Mum's Mood Scale (KMMS) Part 1

Think about the past 7 days, not just how you feel today.

NAME:

DOB:

DATE:

1.	I can sit down and have a good laugh	Yes, always	Yes, sometimes	No, not much	No, never
2.	I look forward for good things to happen	Yes, always	Yes, sometimes	No, not much	No, never
3.	I blame myself when things go wrong	Yes, always	Yes, sometimes	No, not much	No, never
4.	I worry too much and don't know why	Yes, always	Yes, sometimes	No, not much	No, never
5.	I feel frightened and shaky a lot	Yes, always	Yes, sometimes	No, not much	No, never
6.	I can't handle all the stress or I stress out	Yes, always	Yes, sometimes	No, not much	No, never
7.	I feel really no good, like no-one loves me	Yes, always	Yes, sometimes	No, not much	No, never
8.	I can't sleep because I am sad or think too much	Yes, always	Yes, sometimes	No, not much	No, never
9.	I am so sad I have been crying	Yes, always	Yes, sometimes	No, not much	No, never
10.	I think about doing something bad to myself or others	Yes, always	Yes, sometimes	No, not much	No, never
