# Supplementary material for: ‘Having a Quiet Word’: Yarning with Aboriginal Women in the Pilbara Region of Western Australia about Mental Health and Mental Health Screening during the Perinatal Period
Source: Int J Environ Res Public Health. 2019 Nov 1;16(21):4253. doi: 10.3390/ijerph16214253 (PMC6862568; doi:10.3390/ijerph16214253)
Supplement: Supplementary file 1 [file ijerph-16-04253-s001.zip › Supplementary Materials 2.pdf]

Kimberley Mum's Mood Scale Part 2

PART 1 SCORE:

NAME:
DOB:
DATE:

Refer to the KMMS Manual for information on how to facilitate the yarn, guidelines for administration, examples of enquiring/exploring questions for each psychosocial domain, how to determine overall risk, and options for follow-up actions.

EXPLORE THE WOMAN'S STORY, NOTING THE SITUATION AND HER PROTECTIVE AND RISK FACTORS

SUPPORT:

MAJOR STRESSORS:

SELF-ESTEEM / ANXIETY:

RELATIONSHIPS:

CHILDHOOD EXPERIENCES:

SUBSTANCE MISUSE:

SOCIAL, EMOTIONAL AND CULTURAL WELLBEING:

OVERALL RISK OF DEPRESSION AND/OR ANXIETY (PLEASE TICK)

Consider Part 1 score and the risk and protective factors identified during Part 2 in determining overall risk

LOW	Self-care recommended	
MODERATE	Clinical assessment within 1 week	
HIGH	Clinical assessment required within 48–72 hours	
IMMEDIATE CONCERNS	Clinical handover required immediately	

FOLLOW-UP ACTIONS: Explore relevant referrals, or next steps with the woman.
