# Supplementary material for: ‘Having a Quiet Word’: Yarning with Aboriginal Women in the Pilbara Region of Western Australia about Mental Health and Mental Health Screening during the Perinatal Period
Source: Int J Environ Res Public Health. 2019 Nov 1;16(21):4253. doi: 10.3390/ijerph16214253 (PMC6862568; doi:10.3390/ijerph16214253)
Supplement: Supplementary file 1 [file ijerph-16-04253-s001.zip › Supplementary Materials 3.pdf]

Appendix C Questions for each psychosocial domain

Domain	*Psychosocial questions*	
Support	How are you feeling about becoming/being a parent?<br>Do you feel like you have good support around you?	Who are your supports?<br>How are they helping?<br>Do you have someone to talk to about your feelings and your worries?
Major stressors	Are there any things happening in your life right now that are stressing you?<br>Do you have any big worries or losses from the past year that are still worrying/affecting you?	How did/are you coping with these worries/stresses?<br>How are you feeling about giving birth/history of birth experiences/pregnancy?
Self-esteem/anxiety	How are you feeling in yourself?<br>Why do you think you are feeling like that?<br>Do you feel like this just today, or much of the time?<br>Are you worrying a lot or stressing out (feeling anxious)? Can you tell me what gives you those feelings?	How are you managing with everyday things like family, work, home life?<br>Have you been previously diagnosed with depression or anxiety?<br>Have you had medication before to help you manage your depression/anxiety?
Relationships	Having a baby can be a time of big change for everyone, especially the people that you are living with. Who is living in your house?<br>How are they feeling about the pregnancy/baby?<br>Do your family live nearby/with you? Are they supporting you?<br>Has your relationship with the baby's father changed since pregnancy/having the baby?	Do you feel like you have a safe home for you and bub?<br>Have you ever experienced any harmful behaviours from another person? Are these impacting you now?<br>Are you experiencing any jealousy or violence in any of your relationships?<br>Would you like to talk more about any of these things?
Childhood experiences	At this time (pregnancy/young baby) women often think back on their own childhood experiences. There might be good things or hard things that come up. Is there anything you are worrying about? If you would like to talk about anything I am here to listen.	What are some of your happy memories growing up?<br>What were some worries or problems when you were younger?<br>Tell me more about these memories/experiences.<br>Would you like any support to talk about your childhood?
Substance use	Part of keeping you and baby strong is knowing if you are you currently using cigarettes, alcohol or other drugs. Are you currently using any of these?	How has your use changed since being pregnant/having baby?<br>Does your use worry you? How/why?<br>Would you like to get some support and help for these things?
Social and emotional wellbeing	How is your sleeping, eating and physical activity?	You have shared a lot today, thank you. Can you tell me some of the things that keep you strong?
