# Supplementary material for: ‘Having a Quiet Word’: Yarning with Aboriginal Women in the Pilbara Region of Western Australia about Mental Health and Mental Health Screening during the Perinatal Period
Source: Int J Environ Res Public Health. 2019 Nov 1;16(21):4253. doi: 10.3390/ijerph16214253 (PMC6862568; doi:10.3390/ijerph16214253)
Supplement: Supplementary file 1 [file ijerph-16-04253-s001.zip › Supplementary Materials 4.pdf]

Antenatal (Psychosocial) Risk Questionnaire (ANRQ) – Client

V.2004 (Updated 2017) © M-P Austin

The questions below are designed to help you and your clinician understand whether you may benefit from some extra support during this time of change. You may find some questions challenging, but please choose the answers that best apply to you. There are no right or wrong answers.

Please complete all questions, unless instructed to SKIP a question. Once you have completed the questions, your clinician will discuss your responses with you. If you have any concerns about any of the questions, please let your clinician know.

Total ☐

Q1. Have you ever had a period of 2 weeks or more when you felt particularly worried, miserable or depressed?

No	Yes
☐	☐
**If No**, skip to **Q1.c.**	**If Yes**, please answer **Q1.a., Q1.b. and Q1.c.,**

☐

If Yes, did this:

Q1.a. Seriously interfere with your work or your relationships with friends and family?

Not at all	A little	Somewhat	Quite a lot	Very much
☐	☐	☐	☐	☐

☐

Q1.b. Lead you to seek professional help?

No	Yes
☐	☐

Did you see a:
☐ psychiatrist ☐ psychologist/counsellor ☐ GP

If yes, name of professional: ____________________

Did you take tablets/herbal medicine? ☐ No ☐ Yes

If yes, list medication(s): ____________________

☐

Q1.c. Do you have **any other history of mental health problems?** *(e.g. eating disorders, psychosis, bipolar, schizophrenia)* ☐ No ☐ Yes

If yes, list other mental health problems: ____________________

____

Q2. Is your relationship with your partner an emotionally supportive one?

Very much	Quite a lot	Somewhat	A little	Not at all	No partner
☐	☐	☐	☐	☐	☐

☐

Q3. Have you had any stresses, changes or losses *in the last 12 months? (e.g. only: separation, domestic violence, job loss, bereavement etc.)*

No	Yes
☐	☐
**If No**, skip to **Q4.**	**If Yes**, please answer **Q3.a.,**

If yes, please specify: ____________________

☐

If Yes:

Q3.a. How distressed were you by these stresses, changes or losses?

Not at all	A little	Somewhat	Quite a lot	Very much
☐	☐	☐	☐	☐

☐

Q4. Would you generally consider yourself a worrier?

Not at all	A little	Somewhat	Quite a lot	Very much
☐	☐	☐	☐	☐

☐

Q5. In general, do you become upset if you do not have order in your life? *(e.g. regular timetable, tidy house)*

Not at all	A little	Somewhat	Quite a lot	Very much
☐	☐	☐	☐	☐

☐

© M-P Austin. Reproduced with permission. ANRQJune04 (updated May2017).

Antenatal (Psychosocial) Risk Questionnaire (ANRQ) – Client

V.2004 (Updated 2017) © M-P Austin

**Q6.** Do you feel you will have people you can depend on for support with your baby?	Very much ☐	Quite a lot ☐	Somewhat ☐	A little ☐	Not at all ☐		☐

Now you are having a baby, you may be starting to think about your own childhood and what it was like:

**Q7.** Were you emotionally abused *when you were growing up?*	No ☐	Yes ☐					☐
**Q8.** Have you **ever** been sexually ☐ or physically ☐ abused?	No ☐	Yes ☐					☐
**Q9.** *When you were growing up,* did you feel your mother was emotionally supportive of you?	Very much ☐	Quite a lot ☐	Somewhat ☐	A little ☐	Not at all ☐	No Mother ☐	☐

Do you have any other concerns that you would like to talk about today? ______________________________

____

____

© M-P Austin. Reproduced with permission. ANRQJune04 (updated May2017). The Antenatal Risk Questionnaire (ANRQ) was developed by Marie-Paule Austin Chair of Perinatal Mental Health, University of NSW & St John of God Health Care. Reference: Austin, M. P., Colton, J., Priest, S., Reilly, N., & Hadzi-Pavlovic, D. (2013) The Antenatal Risk Questionnaire (ANRQ): Acceptability and use for psychosocial risk assessment in the maternity setting. Women & Birth, 26, 17-25.
